# The Employment of Strychnia in Amaurosis

**Published:** 1842-11

**Authors:** 


*The employment of Strychnia in Amaurosis.*—A laboring boy, 12 years old, received a blow in the right supra-orbital region, by the falling of a pewter vessel which he was endeavoring to remove from a high shelf. At the moment of receiving the blow he perceived a flash of light in the eye, but could see nothing with it afterwards. In three hours he came under the care of Dr. Dusterberg, of Lippstadt. Immediately above the right eye-lid was visible a small blue spot, of the size of a horse-bean. In the eyeball itself nothing abnormal could be detected, no trace of opacity or extravasation of blood. The pupil acted naturally, as in the sound organ, but the power of vision was entirely lost in the right eye; so that he was unconscious when it was directed toward the full glare of the sun. He was treated for two months with bleeding, cold applications, mercurial frictions, blisters, drastics, emetics, electricity, and even the frontal nerve was divided; but all in vain; the amaurosis did not in any degree yield. Subsequently a solution of a grain of nitrate of strychnia in half an ounce of rectified spirit of wine was dropped into the eye four or five times daily: the result of which was, that, in fourteen days, sensations of light were experienced in the affected eye, which, under the continued use of the remedy, increased so that he was enabled to distinguish colored objects. After a period of three months, the power of vision had so far returned that he could recognize bodies at a distance of three feet. At this point the improvement stopped, notwithstanding that the dose of strychnia was increased, and its endemic application had recourse to. The case, however, may be fairly adduced to show the beneficial influence of strychnia on torpid amaurosis.—*Lond. Med. Gaz.*, Aug. 5, 1842, from *Schmidt's Jahrbücher.*

